# Normative prosthodontic care need: does it impact the daily life of young Saudis with high level of oral diseases? A cross sectional study

**DOI:** 10.1186/s12903-017-0418-x

**Published:** 2017-10-23

**Authors:** Fahad Al-Harbi, Maha El Tantawi

**Affiliations:** 1 Department of Substitutive Dental Sciences, College of Dentistry, Imam Abdulrahman Bin Faisal University, P.O.Box 1982, Dammam, 31441 Saudi Arabia; 2 Department of Preventive Dental Sciences, College of Dentistry, Imam Abdulrahman Bin Faisal University, P.O.Box 1982, Dammam, 31441 Saudi Arabia

**Keywords:** Prosthodontic treatment needs, Directed acyclic graphs, Quality of life, Tooth loss, Dental caries

## Abstract

**Background:**

Assessing the need for prosthodontic care previously included older age groups. There is less information about younger populations who may need this care because of high disease levels. The aim of this study was to assess the normative need for prosthodontic care in a young Saudi population with high oral disease levels, the associated factors and its impact on daily life.

**Methods:**

A cross sectional study included Saudi adults in the Eastern Province in 2016. A questionnaire was used to assess personal background (confounders), risk factors affecting oral diseases (exposures) and the impact of oral problems on daily life. A clinical examination assessed tooth loss, the presence of prosthodontic appliances, the presence of untreated decay and need for periodontal care. Directed acyclic graphs identified the confounders to be included in regression models with separate outcomes: normative need for prosthodontic care (binary logistic model) and impact on 6 daily life aspects (ordinal regression models).

**Results:**

Complete data were available for 574/ 700 = 82% and 46.7% needed prosthodontic care with 2 lost teeth on average among adults of mean age = 33.2 years. The confounders controlled for the need for prosthodontic care included socioeconomic status (SES), dental visits last year and health insurance. The confounders for the impact on daily life included age and SES. In adjusted models, normative need for prosthodontic care was significantly associated with untreated decay (OR = 2.09, 95% C.I. = 1.37, 3.19). The impact on daily life was not significantly associated with prosthodontic care need but with untreated decay, especially sleeplessness (regression coefficient = 0.53, 95% C.I. = 0.02, 1.04) and dropping daily activities (regression coefficient = 0.79, 95% C.I. = 0.13, 1.46). In addition, the need for periodontal care was associated with food avoidance (regression coefficient = 0.73, 95% C.I. = 0.28, 1.18).

**Conclusions:**

In Saudi adults in the Eastern Province, there was a considerable normative need for prosthodontic care due to untreated decay. The impact on daily life was related to the underlying oral diseases rather than the need for prosthodontic care itself.

**Electronic supplementary material:**

The online version of this article (10.1186/s12903-017-0418-x) contains supplementary material, which is available to authorized users.

## Background

Dental prosthodontic care includes the provision of dentures whether partial or complete, fixed or removable [1]. Studies have shown that the need for prosthodontic care in adults may follow tooth loss caused by dental caries and periodontal disease [2, 3]. Caries is associated with cariogenic bacteria, sugar exposure, fluoride exposure, toothbrushing, professional cleaning at the dentist’s office, pit and fissure sealant, smoking and personal factors such as age, sex and socioeconomic status (SES). Some of these factors (toothbrushing, professional cleaning, smoking, age, sex and SES) are also associated with periodontitis in addition to other factors including periodontopathic bacteria and diabetes [4]. Obtaining treatment to replace lost teeth is related to health care seeking attitudes and practices such as regular dental visits, the perceived impact of tooth loss on life activities as well as the health care system characteristics including access to care and insurance coverage [3]. In addition, there is an interaction between oral diseases and obtaining care where individuals with advanced disease differ from those at milder stages in the chances of receiving care because of the cost involved and need for dentists with advanced training [5]. On the other hand, obtaining care affects disease detection, progression and treatment and subsequently the risk of tooth loss [6]. The impact that prosthodontic care need has on daily life may be overshadowed by that of the underlying conditions (untreated caries and need for periodontal care) and this may increase as the number of lost teeth increases [1].Thus, the factors associated with the need for prosthodontic care and its impact on daily life are included in a complex network with inter-related factors.

The population of Saudi Arabia is mostly young (median age = 27.2 compared to world median = 30.1 years) [7], with high prevalence of caries [8] and periodontal disease [9] and this may increase the need for prosthodontic care as time passes. Saudis have access to free health care, including dental treatment in governmental facilities under universal coverage [10]. Health insurance is provided by some employers to cover the cost of care in the private sector. Previous studies [1, 11, 12] assessing the need for prosthodontic care were conducted among populations with older age range than the majority of Saudis. Such studies may not apply to Saudis and similar young populations since age is associated with the need for prosthodontic care [13]. There is less information about the need for prosthodontic care in younger populations who are at risk of tooth loss because of high prevalence of oral diseases.

The hypothesis of the present study was that the high level of oral diseases in the studied population would be associated with higher normative need for prosthodontic care and greater impact on daily life. The aims of this study were to assess (1) the normative need for prosthodontic care among adults in the Eastern Province of Saudi Arabia, (2) factors associated with this need and (3) the impact on daily life attributed to the normative need for prosthodontic care compared to that of the underlying oral diseases (untreated decay and need for periodontal care).

## Methods

This study was part of a cross sectional survey conducted from January to May 2016 to assess the oral health status in 5 cities in the Eastern Province of Saudi Arabia (Dammam, Khobar, Dhahran, Saihat and Qatif). The study procedures followed the guidelines of the Helsinki declaration and the ethical approval of the Institutional Review Board of the University of Dammam (IRB- 201702- 048) was obtained. Adults (>18 years old) were recruited using a random sample stratified by sex. Data was collected by the Community Service Unit of the College of Dentistry, University of Dammam in collaboration with community partners. The partners (neighborhood associations) advertised to the community the presence of examiners and the purpose of the survey for a week before the visit to each site. Contact persons from these associations were given the target in each site to ensure that they would reach out to a greater number to make up for potential non-response. At this stage of the survey, 700 adults were targeted. This number was calculated based on the level of prosthodontic treatment needs reported in an earlier study in Jizan, Southern Saudi Arabia (44%) [14] and assuming 10% lower needs among the population in the more affluent Eastern Province.

Data were collected using the Basic Screening Survey methodology of the Association for State and Territorial Dental Directors [15] including clinical examination and questionnaire. The need for prosthodontic care was defined following the description of Abu-Murat et al. [16] as (1) tooth loss without the presence of prosthodontic appliance or (2) the presence of a prosthodontic appliance that was ill fitting or not esthetically acceptable. The clinical examination assessed tooth loss, the number of natural teeth (including root fragments and excluding third molars) and the presence of prosthodontic appliance (fixed or removable, partial or complete on a yes versus no basis in any of the two arches). Only 3.1% of the respondents had existing prosthodontic appliances and none of them was ill fitting or esthetically unacceptable based on clinical examination and feedback from participants. Therefore, in the present study, the need for prosthodontic care was restricted to the first part of the definition [16]. The clinical examination also assessed other oral conditions; namely the presence of untreated decay (defined as a lesion extending into dentin on any tooth detected visually or using the explorer to remove deposits without tactile examination on a yes versus no basis) and the need for periodontal care (if the person needed treatment before the next checkup for conditions that are scored 1–4 by the Community Periodontal Index of Treatment Needs (CPITN) [17] including gingival bleeding, supra or subgingival calculus deposits, periodontal pockets ≥4 mm on a yes versus no basis at individual level).

The standardized English questionnaire [15] was forward translated to Arabic and back translated to English by the investigators to make sure the original meaning was preserved. Two bilingual dentists read the English and Arabic versions of the questionnaire, suggested some changes, retested then ensured the versions were similar. The modified version was pilot tested for clarity on a sample of 30 subjects whose results were not included in the analysis. Their suggested modifications were implemented to develop the final version of the Arabic questionnaire [18]. The questionnaire had three sections:

1. demographic variables; including sex (male or female), age in years and educational level (non-educated, has primary, intermediate, secondary or university education; later recoded into university-educated versus not and used as a socioeconomic (SES) indicator).
2. risk factors for caries and periodontal diseases; including health insurance (yes versus no), dental visits (never, before last year, within last year; later recoded into visited last year versus not), professional cleaning (never, before last year, within last year; later recoded into had cleaning last year versus not), diabetes (none, controlled or uncontrolled; later recoded into yes versus no), smoking (never, former, current; later recoded into yes versus no), brushing habits (twice or more daily, 3+ times weekly, less than that or never; later recoded into brushing twice daily versus not), use of sweetened beverages (daily, weekly, monthly or never; later recoded into daily or less) and topical fluoride exposure (does not use fluoride, uses fluoridated toothpaste, uses other fluoride vehicles including fluoridated mouthwash or professionally applied topical fluoride). We recoded some categories into yes/ no to ensure the presence of marked differences between levels of relevant variables in the analysis investigating the association with the outcome/s.
3. impact of oral diseases on six aspects of daily life including feeling pain, avoiding some foods, feeling embarrassed, being sleepless, skipping work and dropping some daily activities. Participants were asked to indicate if each of these problems occurred most times, sometimes or never.

Subjects were given the questionnaire which began with a brief explanation of the study purpose. There was a consent form which the participants were asked to read and sign in writing. They were seated on a portable dental chair and examined using disposable examination set by the aid of a portable dental light on a stand. Three examiners were trained and calibrated to a gold standard examiner to an acceptable level of agreement (Kappa ≥0.6) on 20 subjects whose examination results were not included in the analysis. Participants were examined and referred to receive treatment to the clinics of the College of Dentistry, University of Dammam.

### Conceptual framework

**Fig. 1:**
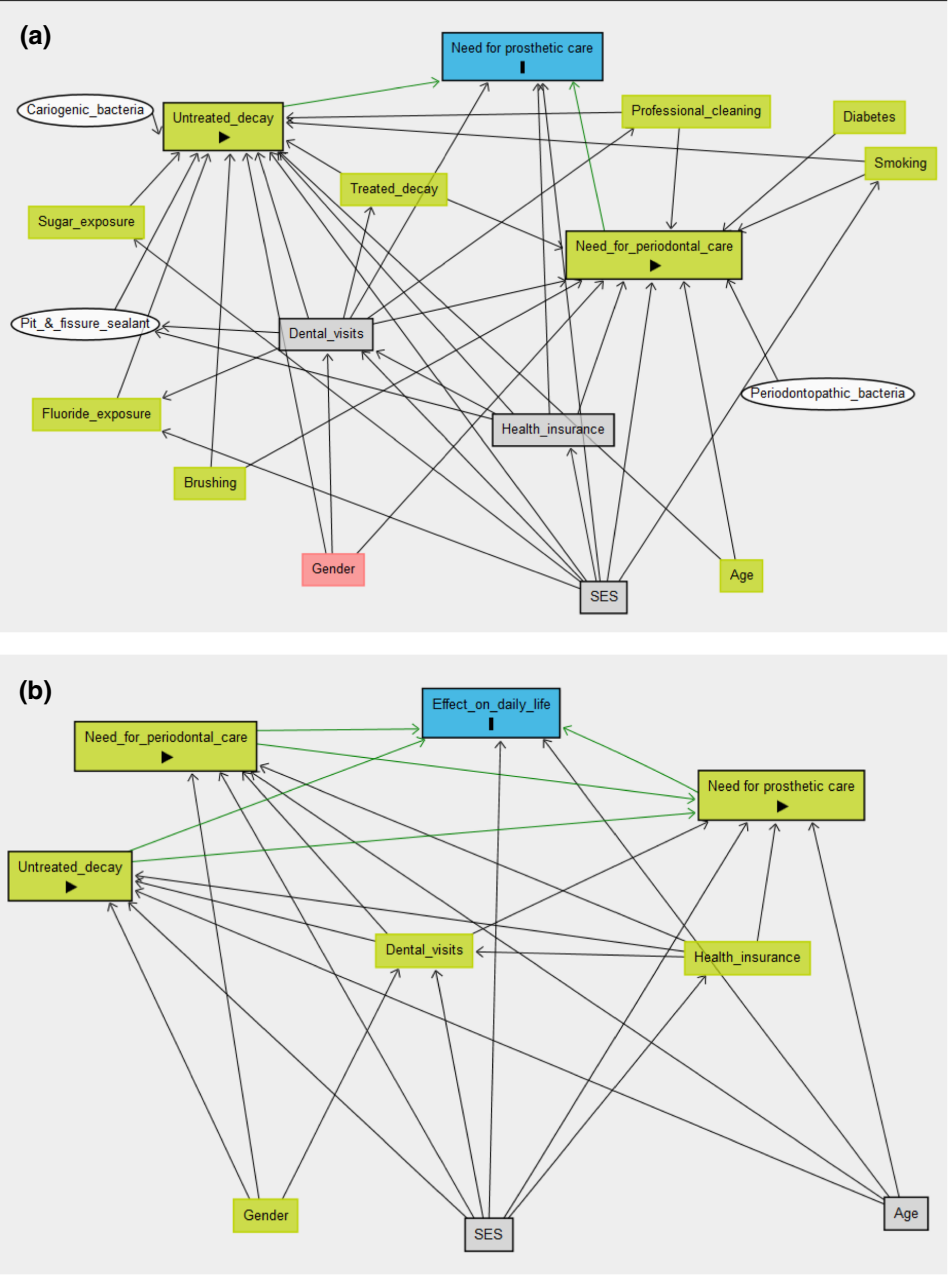
**a**) DAG of need for prosthodontic care (outcome; blue rectangle with black frame) and untreated decay and need for periodontal care (exposures; green rectangles with black frame). SES, dental visits and health insurance are the confounders to be controlled in the multivariable model (grey rectangles), (**b**) DAG of daily life (outcome; blue rectangle with black frame) and need for prosthodontic care, untreated decay, need for periodontal care (exposures; green rectangles with black frame). SES and age are the confounders to be controlled (grey rectangles). The figures include other variables with potential relationships to the outcomes which were measured in the study (other green rectangles) or not (white ovals)

We developed a framework (Additional file 1) to visualize the relationship between the study variables using Directed Acyclic Graphs (DAGs) (Fig. 1a and b) plotted by Dagitty (http://dagitty.net/) [19]. In the 1st framework, the outcome was the need for prosthodontic care and the exposures were untreated decay and need for periodontal care. Several factors were included because they were related to these two oral diseases and were divided into practices that increase their risk and demographic factors. The other framework showed the association between the impact on daily life and need for prosthodontic care in addition to untreated decay and need for periodontal care. In these DAGs, the relationships were marked by arrows that proceeded only in one direction from exposure to outcome, hence the term “directed” and did not loop back in the other direction, hence the term “acyclic” [20].

Confounders were detected using the software algorithm and a set of them was selected containing the minimal number of variables that needed to be controlled in multivariable analysis to produce unbiased estimates of the association between the outcomes and exposures [20].

### Analysis

The study had two outcomes:

1. The need for prosthodontic care (yes or no in binary logistic regression). The exposures were untreated decay and need for periodontal care and confounders were identified by DAG. Univariate regression models were developed to include each exposure/ confounder followed by a multivariable model controlling for the identified confounders.
2. The impact on six aspects of daily life (most times, sometime or never in ordinal regression). The exposures were need for prosthodontic care, untreated decay and need for periodontal care. These were included in multivariable models for each of the six aspects of impact on daily life controlling for the confounders detected by the DAG and adjusted for multiple comparisons across the six outcomes.

Odds ratios/regression coefficients and 95% confidence intervals were calculated. Statistical analysis was performed using SPSS version 20.0. *P* values <0.05 were considered statistically significant.

## Results

Out of the 700 adults targeted, complete data were available for 574 (82%). Of the 126 with incomplete data, 44 refused clinical examination, 37 did not fill the questionnaire, 23 partly filled the questionnaires omitting key variables and 22 wanted only to be examined for referral and had incomplete examination forms and questionnaires. The comparison of participants with missing data and those who were included in the analysis showed no significant differences in available variables and no evidence of selection bias. The mean (SD) age of the participants was 33.2 (11.4) with 72.3% <40 years old (Table 1). Males represented 46.9% and 33.4% of the respondents were university-educated. Those who brushed twice daily represented 56.3% whereas 38.5% used no fluoride products and 47.6% had sweetened beverages daily. Current smokers were 22.8% and 10.3% had diabetes. Of all participants, 47.6% had visited the dentist last year, 25.4% had their teeth professionally cleaned by dentist last year and 29.3% had health insurance.

**Table 1:** Personal background, risk factors for oral diseases and need for care in adults from the Eastern Province, Saudi Arabia, 2016 (*n* = 574)

Age: mean (SD)		33.2 (11.4)
Males: n (%)		269 (46.9)
University educated: *n* (%)		192 (33.4)
Brushing twice daily: *n* (%)		323 (56.3)
Fluoride exposure: *n* (%)	None	221 (38.5)
	Toothpaste	335 (58.4)
	Other fluoride measures	18 (3.1)
Daily use of sweetened drinks: *n* (%)		273 (47.6)
Current smoking: *n* (%)		131 (22.8)
Has diabetes: *n* (%)		59 (10.3)
Visited the dentist last year: *n* (%)		273 (47.6)
Had professional tooth cleaning last year: *n* (%)		146 (25.4)
Has health insurance: *n* (%)		168 (29.3)
Has tooth loss: n (%)		272 (47.4)
Number of lost teeth: mean (SD)		2.0 (3.9)
Has prosthodontic appliance: *n* (%)	Maxillary	15 (2.6)
	Mandibular	3 (0.5)
	None	556 (96.9)
Has untreated decay: *n* (%)		408 (71.1)
Has need for periodontal care: *n* (%)		382 (66.6)
Has need for prosthodontic care: *n* (%)		268 (46.7)

Clinical examination showed that 47.4% have lost at least one tooth with an average number of 2 lost teeth. Most participants (96.9%) had no prosthodontic appliances, 71.1% had untreated decay, 66.6% needed periodontal care and 46.7% needed prosthodontic care.

Table 2 shows that at least 68.7% of participants never felt embarrassed or sleepless, skipped work or dropped daily activities because of oral problems. However, most times 15.9% felt pain and 14.8% avoided some foods because of oral problems.

**Table 2:** Frequency of impact of oral diseases on six aspects of daily life

	Never *N* (%)	Sometimes *N* (%)	Most times *N* (%)
Feeling pain	220 (38.4)	262 (45.7)	91 (15.9)
Avoiding food	294 (51.2)	195 (34)	85 (14.8)
Feeling embarrassed	433 (75.5)	84 (14.7)	56 (9.8)
Sleepless	394 (68.7)	113 (19.7)	67 (11.6)
Skipping work	490 (85.3)	59 (10.3)	25 (4.3)
Dropping activities	459 (80)	89 (15.5)	26 (4.5)

The DAG (Fig. 1 and b) showed that the confounders to be controlled in the model of need for prosthodontic care were dental visits, health insurance and SES. The confounders to be controlled in the model where the outcomes were aspects of the impact on daily life were SES and age.

Table 3 shows that in univariate regression, university-educated persons had significantly lower odds of need for prosthodontic care compared to non-university educated persons (OR = 0.58, 95% C.I. = 0.40, 0.84). Untreated decay was associated with significantly higher odds of need for prosthodontic care (OR = 1.79, 95% C.I. = 1.24, 2.59) and so was the need for periodontal care (OR = 1.72, 95% C.I. = 1.21, 2.45). In the multivariable model including the confounders selected by the DAG, only untreated decay was significantly associated with higher odds of need for prosthodontic care, OR = 2.09, 95% C.I. = 1.37, 3.19 while the need for periodontal care was not significantly associated (OR = 1.49, 95% C.I. = 0.99, 2.25).

**Table 3:** Impact of untreated decay and need for periodontal care on need for prosthodontic care

	UOR (95% C.I.)	*P* value	AOR (95% C.I.)	*P* value
University-educated	0.58 (0.40, 0.84)*	0.004*	0.62 (0.41, 0.93)*	0.02*
Dental visits last year	1.08 (0.77, 1.50)	0.66	1.16 (0.80, 1.69)	0.44
Health insurance	1.23 (0.85, 1.77)	0.27	0.87 (0.57, 1.33)	0.52
Untreated decay	1.79 (1.24, 2.59)*	0.002*	2.09 (1.37, 3.19)*	0.001*
Need for periodontal care	1.72 (1.21, 2.45)*	0.003*	1.49 (0.99, 2.25)	0.06

*UOR* unadjusted odds ratio in univariate models, *AOR* adjusted odds ratio in multivariable model controlled for confounders, *C.I.* confidence interval

*: statistically significant at *p* < 0.05. Multivariable model correctly classified 64.4% of participants

Table 4 shows that the unadjusted need for prosthodontic care was associated with significantly higher likelihood of frequent feeling of pain (regression coefficient = 0.37, 95% C.I. = 0.05, 0.69). In the multivariable models, when the confounders identified by the DAG were controlled, the need for prosthodontic care was not significantly associated with any of the aspects of daily life. In this multivariable model, untreated decay was associated with significantly higher likelihood of frequent sleeplessness (regression coefficient = 0.53, 95% C.I. = 0.02, 1.04) and dropping daily activities (regression coefficient = 0.79, 95% C.I. = 0.13, 1.46). The need for periodontal care was associated with significantly higher likelihood of frequent avoidance of foods (regression coefficient = 0.73, 95% C.I. = 0.28, 1.18). The confounders had some significant associations with the outcomes; increasing age was significantly associated with less frequent feeling of pain (regression coefficient = −0.02, 95% C.I. = −0.04, −0.01) but not with any other aspect. University-educated persons had significantly less likelihood of sleeplessness (regression coefficient = −0.83, 95% C.I. = −1.32, −0.34) with no association with any other aspect.

**Table 4:** Impact of need for prosthodontic care (unadjusted and adjusted), untreated decay and need for periodontal care on daily life aspects

Factor	Regression coefficient (95% C.I.)					
	Feeling pain	Avoiding foods	Embarrassment	Sleeplessness	Skipping work	Dropping daily activities
Unadjusted need for prosthodontic care	0.37 (0.05, 0.69)*	0.27 (−0.06, 0.60)	0.09 (−0.32, 0.50)	0.22 (−0.15, 0.58)	0.39 (−0.12, 0.89)	0.18 (−0.26, 0.62)
Untreated decay	0.38 (−0.06, 0.81)	0.20 (−0.65, 0.25)	0.24 (−0.79, 0.32)	0.53 (0.02, 1.04)*	0.38 (−0.36, 1.11)	0.79 (0.13, 1.46)*
Need for periodontal care	0.02 (−0.44, 0.40)	0.73 (0.28, 1.18)*	−0.19 (−0.34, 0.72)	0.003 (−0.48, 0.49)	0.59 (−0.16, 1.34)	0.07 (−0.53, 0.67)
Need for prosthodontic care	−0.03 (−0.43, 0.49)	0.004 (−0.48, 0.47)	0.01 (−0.59, 0.57)	0.14 (−0.38, 0.65)	0.16 (−0.56, 0.87)	0.12 (−0.51, 0.75)

*C.I.* confidence interval, *: statistically significant at *p* < 0.05

## Discussion

Our study showed that 46.7% of the study sample who were Saudi adults in the Eastern Province needed prosthodontic care. After controlling for confounders, the odds of normative need for prosthodontic care were double in those with untreated decay and were not significantly associated with the need for periodontal care. When adjusted for untreated decay, need for periodontal care and identified confounders, the need for prosthodontic care did not significantly impact daily life. Our findings thus partly support the study hypothesis where higher disease level was associated with higher normative need for prosthodontic care but not with impact on daily life.

Our study was cross sectional; a design that is challenged by biases and confounders [21]. DAGs help address some of these issues by identifying variables to adjust and by guiding model construction [22]. They helped us identify the confounders to control while developing regression models to test the associations included in the framework. Previously, DAG was used to identify the confounders for a multivariable model assessing causality between the presence of unreplaced lost teeth and higher risk of mortality [23]. It was also used to investigate the association between parental and participant’s paan chewing controlling for age, religious beliefs and parenting behaviours [24].

In our study, the proportion of those in need of prosthodontic care was lower than that previously reported in Southern Saudi Arabia (56.8%) [14] which might be due to methodologic or actual differences in need for care. Our results showed that almost all those with tooth loss (47.4%) needed prosthodontic care (46.7%) indicating that they did not seek care to replace their loss. The difference between the examiner-assessed normative need and the perceived need expressed and later acted upon by individuals was similarly reported in previous studies [1, 25]. This difference may be attributed to people with few lost teeth being less likely to replace them [11] or to cost being a barrier for seeking treatment [26]. The need for prosthodontic care may be defined clinically based on any loss of teeth without replacement [16] or the presence of specific number of occlusal units with and without prosthodontic replacement [27]. Another perspective of the definition of prosthodontic need for care includes normative and perceived needs. This draws attention to the importance of incorporating patient-based outcomes in studies assessing community needs and evaluating treatment interventions. It is also useful to consider these differences during dental workforce planning especially in the public sector where services are provided free of charges.

In the present study, adults with untreated decay had double the odds of need for prosthodontic care as those without untreated decay. This agrees with Correa et al. who reported that caries at age 15 was significantly associated with prosthodontic needs at age 24 (prevalence ratio = 2.9) [28]. The normative need for prosthodontic care in our study was not significantly associated with the need for periodontal care. This disagrees with previous studies reporting that gingival bleeding was a predictor of unreplaced lost teeth [29] and that gingival attachment loss predicted multiple unrestored tooth loss [30]. The difference between our results and these studies may be attributed to the fact that they included older participants than those in our study. Periodontal disease, usually taking longer time to develop and to progress than caries, might be expected to affect the need for prosthodontic care at older age.

Within the context of our study setting and the characteristics we studied, there was no association between health care system factors and the normative need for prosthodontic care which was significantly associated neither with previous dental visits nor health insurance coverage. This lack of association might be because we focused on dental visits in the previous year as an indicator of recent care. It is recommended that future studies assess the effect of regular care as indicated by periodic checkups versus visiting on pain. Similarly, the non- significant association with health insurance might be attributed to the presence of universal coverage for health care in governmental facilities in Saudi Arabia. Insurance may have greater impact in privately financed health care systems through alleviating the problem of cost [31]. Our finding disagrees with Davidson et al. who reported an increase in the volume of prosthodontic treatment delivered to Swedes younger than 65 years when the reimbursement system was changed to cover these services [32].

In the present study, normative need for prosthodontic care seemed to be related to lower SES where university education was associated with less need for care. This agrees with a study reporting that adult Greeks with highest education had significantly lower odds of prosthodontic care need whether they were middle aged or older (OR = 0.21 and 0.51) [12]. This association in our study was independent of receiving dental care and of the presence of oral diseases. Further investigation is needed to understand the mechanism by which SES is associated with the normative need for prosthodontic care in this and similar populations.

In our study, prosthodontic care need was not independently associated with daily life aspects although some association was attributed to the underlying diseases. This finding forms the basis for rejecting the related part of the study hypothesis and shows that in younger age groups, the mere loss of few teeth does not negatively impact life. Our finding agrees with Tan et al. who showed that it was possible to maintain oral function and quality of life when few teeth were lost and not replaced [27]. On the contrary, our results differ from a study conducted among older participants that reported significantly greater impact on daily life among adult Brazilians with need for prosthodontic treatment in the maxillary or mandibular jaws (adjusted risk prevalence = 1.29 and 1.34) [33]. Our findings also disagree with Azevedo et al. who reported that there was an association between impact on daily life and more detailed measurement of need for prosthodontic care than we used in our study including intra oral location and number of units to be replaced [1]. These differences between our findings and the other studies highlight the importance of generalizing results to populations with similar age profiles.

The participants in our study were similar in age to the national Saudi profile [7], with more university education (33.4% vs 21.8%) [34] and dental visits last year (47.6% vs 30.1%) [35], with similarly high levels of caries [8], periodontal disease [9] and tooth loss [36]. Our results can thus be generalized to young populations with high levels of oral diseases in spite of the accessibility of health care services and universal coverage of health care.

The present study is limited by its cross sectional design and it is recommended that future longitudinal studies focus on the impact of the two oral diseases on future need of prosthodontic care and how this is affected by the use of less costly treatment alternatives such as the shortened dental arch approach. Self-reporting might have been affected by social desirability bias resulting in overestimation of positive practices such as brushing and dental visits [37].

## Conclusions

Our study showed that in younger populations with high levels of oral diseases, the need for prosthodontic care was considerable and was associated with untreated decay. Daily life was not negatively impacted by this need for prosthodontic care but by untreated decay and need for periodontal care. These results indicate the importance of prioritizing health care resources to prevent and control oral diseases before delivering rehabilitative prosthodontic services to improve the quality of life of younger populations.

## Additional file

Additional file 1: Code for framework 1, code for framework 2. This file includes the code for DAGs which were copied (each one separately) and pasted into the “Model code” box at http://dagitty.net/dags.html# to plot the DAGs. (DOCX 14 kb)

## Abbreviations

CPITN: Community Periodontal Index of Treatment Needs
DAG: Directed Acyclic Graph
IRB: Institutional Review Board
SES: Socioeconomic status
