# Supplementary material for: Normative prosthodontic care need: does it impact the daily life of young Saudis with high level of oral diseases? A cross sectional study
Source: BMC Oral Health. 2017 Oct 23;17:128. doi: 10.1186/s12903-017-0418-x (PMC5653980; doi:10.1186/s12903-017-0418-x)
Supplement: Additional file 1: — Code for framework 1, code for framework 2. This file includes the code for DAGs which were copied (each one separately) and pasted into the “Model code” box at http://dagitty.net/dags.html# to plot the DAGs. (DOCX 14 kb) [file 12903_2017_418_MOESM1_ESM.docx]

Codes for DAGs are copied (each one separately) and pasted into the “Model code” box at <http://dagitty.net/dags.html#> to plot the DAGs

**Framework 1**

Age 1 @3.815,3.080

Brushing 1 @-2.257,1.554

Cariogenic_bacteria U @-3.678,-4.600

Dental_visits A @-0.930,-0.904

Diabetes 1 @5.277,-4.486

Fluoride_exposure 1 @-3.549,0.418

Gender 1 @-0.848,2.978

Health_insurance A @1.666,0.673

Need%20for%20prosthetic%20care O @1.313,-5.617

Need_for_periodontal_care E @2.817,-2.345

Periodontopathic_bacteria U @4.801,0.079

Pit_%26_fissure_sealant U @-3.678,-1.074

Professional_cleaning 1 @3.298,-4.583

SES A @2.241,3.436

Smoking 1 @5.634,-3.723

Sugar_exposure 1 @-3.666,-2.752

Treated_decay 1 @-0.155,-3.294

Untreated_decay E @-2.104,-4.464

Age Need_for_periodontal_care Untreated_decay

Brushing Need_for_periodontal_care Untreated_decay

Cariogenic_bacteria Untreated_decay

Dental_visits Fluoride_exposure Need%20for%20prosthetic%20care Need_for_periodontal_care Pit_%26_fissure_sealant Professional_cleaning Treated_decay Untreated_decay

Diabetes Need_for_periodontal_care

Fluoride_exposure Untreated_decay

Gender Dental_visits Need_for_periodontal_care Untreated_decay

Health_insurance Dental_visits Need%20for%20prosthetic%20care Need_for_periodontal_care Pit_%26_fissure_sealant Untreated_decay

Need_for_periodontal_care Need%20for%20prosthetic%20care

Periodontopathic_bacteria Need_for_periodontal_care

Pit_%26_fissure_sealant Untreated_decay

Professional_cleaning Need_for_periodontal_care Untreated_decay

SES Dental_visits Fluoride_exposure Health_insurance Need%20for%20prosthetic%20care Need_for_periodontal_care Smoking Sugar_exposure Untreated_decay

Smoking Need_for_periodontal_care Untreated_decay

Sugar_exposure Untreated_decay

Treated_decay Need_for_periodontal_care Untreated_decay

Untreated_decay Need%20for%20prosthetic%20care

**Framework 2**

Age A @3.516,3.029

Dental_visits 1 @-0.359,-1.620

Effect_on_daily_life O @0.180,-5.966

Gender 1 @-1.635,3.063

Health_insurance 1 @2.240,-1.489

Need%20for%20prosthetic%20care E @2.513,-4.514

Need_for_periodontal_care E @-2.436,-5.378

SES A @0.162,3.402

Untreated_decay E @-3.205,-3.086

Age Effect_on_daily_life Need%20for%20prosthetic%20care Need_for_periodontal_care Untreated_decay

Dental_visits Need%20for%20prosthetic%20care Need_for_periodontal_care Untreated_decay

Gender Dental_visits Need_for_periodontal_care Untreated_decay

Health_insurance Dental_visits Need%20for%20prosthetic%20care Need_for_periodontal_care Untreated_decay

Need%20for%20prosthetic%20care Effect_on_daily_life @1.445,-4.890

Need_for_periodontal_care Effect_on_daily_life Need%20for%20prosthetic%20care

SES Dental_visits Effect_on_daily_life Health_insurance Need%20for%20prosthetic%20care Need_for_periodontal_care Untreated_decay

Untreated_decay Effect_on_daily_life Need%20for%20prosthetic%20care
